# The Prevalent Fever of Cherokee-Georgia

**Published:** 1873-04

**Authors:** Robert Battey

**Affiliations:** Georgia


### THE PREVALENT FEVER OF CHEROKEE-GEORGIA.

BY ROBERT BATTEY, M.D., OF GEORGIA,

One of the most common diseases—certainly the most common febrile affection—which physicians in the Cherokee region of Georgia are called upon to treat, is a fever of composite type, which would likely be classed by Professor Flint under the head of typho-malarial fever. It is, however, no part of my purpose to enter into a discussion of its proper classification; nor, indeed, to treat of the fever in any methodical way. I desire, at the outset of what I have to say upon the subject, to disclaim any intention *to teach anybody anything* as to the classification, etiology, diagnosis, prognosis or treatment of the malady. In no sense can I arrogate to myself either the airs or the sentiments of an *authority* upon this subject.

In retrospecting my past experience with the particular mixed type of fever to which I allude, there are two facts which stand prominently before me—first, that the *mortality* of the disease, under my management, has markedly diminished of late years; and, secondly, that my methods of *treatment* have undergone a corresponding change.

That the lessened mortality is linked, in my mind, intimately with the change in treatment, I will not deny, and yet I shall not insist upon this view with any who may be disposed to gainsay it. I purpose only to put before my brethren the two points in my experience, and let those whose success in the malady is not quite what they would like to have it, draw their own conclusions. If the labor of this writing shall favorably determine the issue of but *one human life*, my object will have been fully accomplished, and the labor richly repaid.

The Cherokee country of Georgia, as it is called, is intermediate in latitude and climate between the typhoid region of the more northern States and the decidedly malarial region which borders upon the gulf. Our morbific influences are like in kind to both, and different in degree from either of those regions. In the malady under consideration, we may, I think, fairly recognize the co-existence, in ever-varying proportions, of the two characteristic disease-types of the typhoid and malarial districts. It is but rarely, as it seems to me, that we see pure, uncomplicated cases of either typhoid or malarial remittent in Cherokee-Georgia.

The particular class of cases to which I now have reference present somewhat diversified symptoms, but still enough of common points of resemblance to mark definitely this commingling of a typhoid element with a malarial element, and pass, for the most part, under the general designation of remittent fever, as generally understood here.

The pulse, in the great majority of instances, ranges, in the earlier stages of the malady, between one hundred and one hundred and twelve beats per minute. While this moderate frequency of the pulse is for days quite uniformly the same both morning and evening, there are well-marked evidences of *remission* in the fever, as shown by change of temperature and by alternate exacerbation and subsidence of the nervous symptoms. *There is an almost uniform sensitiveness of the bowels to the action of purgatives.* My patient tells me that his bowels were a little bound—that he took his customary purgative and found the purging more frrquent and copious than he expected; and at the same time there is irritability of the stomach and, very commonly, more or less bilious vomiting. *Quinine does not control the fever,* in this stage, nor diminish the frequency of the pulse, but, on the contrary, usually elevates both, and markedly increases the headache and irritability of stomach. *Mercurials act harshly upon the bowels,* induce diarrhœa, and also irritate more or less the stomach. The duration of the disease, under my present treatment, varies from three days to two weeks, and rarely three or four weeks. So much by way of designating the class of cases.

It was formerly my habit to treat these cases moderately with calomel, and decidedly with quinine—and, as I have already remarked, with a success not quite satisfactory; indeed, with an attendant mortality certainly very much out of proportion to that which now attends my ministrations in similar cases. It was my experience in the use of these remedies that my early doubts of diagnosis were not unfrequently soon resolved by the unmistakable symptoms of a typhoid state difficult to manage; and I consoled myself *then* with the belief that it was so foreordained, but *now* I think I can see it differently.

At present I discard the mercurials *entirely* from my armamentarium, and use quinine only at certain periods, and then tentatively and with misgivings of its general utility. I may also remark that I have witnessed nothing in the results of veratrum which has tempted me to rely at all upon it, or to go on with its use.

I avow unhesitatingly my disbelief in disease as an *entity,* which is to be expelled *vi et armis* from the human system, but I decline to commit myself either to an acceptance or rejection of the theory of actual disease-germs conveyed through the vessels with the vital fluid. It is enough, for the present, that I express my conviction that *useful results* do grow out of the treatment of typho-malarial fever as though we did actually believe that separate germs of both typhoid and malarial poisons, in varying proportions, were actually floating in the blood, and exercising an irritating and inflaming action both upon the general economy and upon certain special, vital organs, which, by reason of idiosyncrasy or otherwise in each particular case, manifest a deficiency of resisting power. It is useful to treat it as though we did really believe that there rests in the human organism a power, when suitably assisted, of expelling from the circulating fluid these morbific germs through the excretions.

In this *practical* belief I do, in the disease under consideration, administer so-called eliminating remedies, and endeavor "to support the powers of life."

I purge the bowels *gently*, when it has not already been done, and when the canal has once been well cleared, abandon purgatives and content myself with laxative enemata. For the most part, the *citrate of potassa* in the form of neutral mixture, and *muriatic acid* in doses of two to five drops every two hours in the form of lemonade, are my reliance as eliminating and supporting agents, with proper diet.

It is my custom, when I cannot conveniently do it myself, to have the pulse counted by the watch *early every morning.* When the pulse is found at, or very nearly, the normal standard of the individual, quinine is given, but at no other time and under no other circumstances. I have often omitted the quinine altogether, and this when the pulse, as I have said, indicated it, and have not found the paroxysm to return. But in other instances it does return, and this for several successive days. I therefore esteem it of service. I never give the quinine upon an irritable or inflamed stomach to be vomited back. If there be much headache, whatever the pulse, I esteem quinine to be contra-indicated.

In addition to these measures, I very uniformly give Dover's powder, and discontinue other medicines at night, that my patient may have undisturbed repose. Headache and other nervous symptoms are met by the administration of bromide of potassium as needed.

The complications which have chiefly required attention have been found most frequently in the stomach, next in the brain, and lastly in the liver; the slight pulmonary trouble, when present, has required no special treatment. If the case prove unusually obstinate, and particularly if the pulse run unusually high, a careful scrutiny will ordinarily discover the cause in one of these organs. Under such circumstances, I have often known the pulse to drop twenty beats per minute *immediately* upon the drawing of a large blister—and it is upon this that I chiefly rely to remove such collateral troubles.

It is foreign to my purpose to argue at all the principles upon which this treatment is based. I give it simply as the result of my experience with the disease, and coupled with the fact that it is at least coincident with a marked diminution in the mortality of the disease in my hands.

ROME, GEORGIA, February 25, 1873.

